# Clinical and Radiographic Evaluation of Plasma Rich in Growth Factors (PRGF) for Alveolar Ridge Preservation in the Anterior Maxilla

**DOI:** 10.3390/jfb17090452

**Published:** 2026-09-07

**Authors:** Tejas Pande, Sourav Panda, Anurag Satpathy, Abhaya Chandra Das, Subharchana Das, Aditya Kute

**Affiliations:** Department of Periodontics, Institute of Dental Sciences, Siksha ‘O’ Anusandhan University, Bhubaneswar 751003, Odisha, India; dr.tejaspande@gmail.com (T.P.); anuragsatpathy@soa.ac.in (A.S.); abhayadas@soa.ac.in (A.C.D.); subharchana97@gmail.com (S.D.); adityakute95@gmail.com (A.K.)

**Keywords:** Plasma Rich in Growth Factors (PRGF), alveolar ridge preservation, socket healing, anterior maxilla, tooth extraction, spontaneous healing

## Abstract

Tooth extraction followed by alveolar ridge resorption may compromise the aesthetics and future implant placement, especially in the anterior maxilla. This comparative clinical study assessed the effectiveness of Plasma Rich in Growth Factors (PRGF) for alveolar socket preservation compared with Spontaneous Healing (SH). Twenty extraction sockets were allocated to either of the groups (*n* = 10 each) and followed for 3 months. Both groups demonstrated dimensional changes following extraction;however, PRGF-treated sockets showed significantly better preservation of the horizontal ridge width at the crest, at 1 mm below the crest, and at 3 mm below the crest compared with SH (all *p* < 0.001). The PRGF group also demonstrated a significantly smaller decrease in the socket depth and a greater increase in the radiographic bone density (both *p* < 0.001). Early healing, soft tissue healing, and postoperative pain outcomes were significantly more favorable in the PRGF group during the follow-up period. Histomorphometricanalysis showed no significant difference between the PRGF and SH groups in terms of percentage of new bone formation and connective/ fibrovascular tissue.Though dimensional changes could not be completely prevented, PRGF significantly decreased the post-extraction resorption and improved the tissue quality, signifying its potential as a biologically driven approach for alveolar ridge preservation in the anterior maxilla.

## 1. Introduction

Alveolar bone is a tooth-dependent, specialized structure that develops with tooth eruption and undergoes changes after functional loading. It consists of alveolar bone proper and the supporting alveolar bone, including cortical plates and cancellous bone [1]. The bundle bone lines the socket and consists of Sharpey’s fibers inserting from the periodontal ligament. It plays a major role in maintaining alveolar integrity. The existence of the bundle bone is dependent on the presence of the tooth [2]. Following tooth extraction, the loss of the periodontal ligament-mediated stimulation causes a rapid resorption of the bundle bone, starting a cascade of dimensional changes in the alveolar ridge [3]. These changes are mainly seen at the crestal region, which is mostly composed of bundle bone and undergoes early osteoclastic remodeling. This resultant resorption plays a role in the horizontal ridge contraction and vertical loss of the ridge height [4]. The anterior maxilla is mostly exposed to these changes because of the presence of thin buccal cortical plates and a relatively higher proportion of cancellous bone. Even though this anatomical structure supports a rich vascular supply and favorable healing, it also makes the region more prone to rapid dimensional collapse, especially on the facial aspect [5]. These changes have significant clinical implications, mainly in the aesthetic areas, and where the preservation of the ridge contour is important for an ideal prosthetic and implant outcome [6].

Immediately after tooth extraction, the sockets undergo a sequence of hemostasis, inflammation, proliferation, and remodeling. Initially, a blood clot forms within the socket, which serves as a provisional matrix rich in platelets and growth factors [7]. This is then followed by an infiltration of inflammatory cells, angiogenesis, and then granulation tissue formation. Woven bone is then deposited by osteogenic cells, which is eventually remodeled into a lamellar type of bone over time [8]. Many molecular and cellular events like angiogenesis, fibroblast proliferation, and extracellular matrix deposition regulate socket healing and determine the quality of newly formed bone [9]. Spontaneous healing does not prevent this dimensional reduction of the alveolar ridge and instead bone regeneration happens within a progressively contracting osseous envelope, leading to a net loss of ridge width and height [10]. It has been stated that a loss of 50% of the ridge width may occur within the first year after extraction, with most of the resorption happening in the initial three months. Horizontal bone loss in the buccal aspect is more prominent than vertical bone loss [4,11]. Existing studies based on Cone Beam Computed Tomography (CBCT) have also suggested that the majority of dimensional changes happen within the first 3–6 months after extraction, especially in areas with thin buccal cortical plates [12,13]. These changes can compromise future implant placement by impacting implant positioning, primary stability, and aesthetic outcomes. Thus, approaches aimed at reducing post-extraction resorption have become an important component of implant therapy, especially in the anterior maxilla [14].

To maintain space and support bone regeneration, alveolar ridge preservation techniques rely on the placement of graft materials, often along with barrier membranes [15]. These approaches have shown reduced dimensional changes and are mainly based on the osteoconductive principle [14,16]. However, they may be correlated with limitations such as residual graft particles and variable rates of new vital bone formation [17]. Recently, there has been an increase in biologically driven regenerative strategies that aim to increase the natural healing cascade instead of just relying solely on mechanical space maintenance. Autologous platelet concentrates (APCs) have emerged as a potential biomaterial because of their ability to release a concentrated source of growth factors involved in tissue regeneration [18]. PRGF is characterized by controlled platelet activation and a leukocyte-poor composition. It forms a structured fibrin scaffold that supports cell migration and allows sustained release of growth factors like Platelet-Derived Growth Factor (PDGF), Transforming growth factor-β (TGF-β), Vascular Endothelial Growth Factor (VEGF), and Insulin-like Growth Factor [19,20]. These factors play a major role in angiogenesis, osteoblast proliferation and extracellular matrix formation [21,22,23,24]. Moreover, PRGF provides a fibrin scaffold that enhances cellular migration and facilitates early wound healing [25].

PRGF does not function through space maintenance, unlike particulate graft materials, but rather through biological stimulation of the intrinsic healing processes. Its leukocyte-poor composition may further reduce the inflammatory response, minimizing the early crestal bone loss [26,27,28]. Even though previous research has suggested benefits of platelet concentrates on soft tissue healing and bone regeneration, the evidence about their effectiveness as a solitary biomaterial for alveolar ridge preservation remains limited [27,28,29,30,31].

There is a scarcity of clinical studies that directly compare PRGF with spontaneous healing, especially in the anterior maxilla region, where dimensional stability is important. Hence, thepresent study aims to check the clinical effectiveness of PRGF when compared to spontaneous healing for alveolar ridge preservation in the anterior maxilla.

## 2. Materials and Methods

### 2.1. Study Design

This prospective, non-randomized, comparative clinical study was conducted at the Department of Periodontics and Oral Implantology, Institute of Dental Sciences, Siksha ‘O’Anusandhan (Deemed to be University), Bhubaneswar, Odisha, India, between November 2024 and March 2026. The study was performed in accordance with the Declaration of Helsinki (1975, revised in 2000). Ethical approval was obtained from the Institutional Ethics Committee of the Institute of Dental Sciences, SOA (Deemed to be University) (IEC-IDS/IDS/SOA/2024/III-19). Written informed consent was obtained from all participants before enrolment. The study protocol was registered on 26.08.2026 at ISRCTN (UK Clinical Registry) with Registration No.ISRCTN18155743. The study was registered retrospectively because trial registration was inadvertently not completed before recruitment. The study objectives, eligibility criteria, intervention, and outcomes had been pre-specified in the ethics-approved protocol.

### 2.2. Study Population

Male and female patients aged ≥18 years with an indication for uncomplicated tooth extraction in the anterior maxilla were included in the study. A minimum socket depth of 7 mm was required. Willingness of the patients toattend follow-up visits was also a requisite for the inclusion criteria. Participants with a presence of dehiscence, loss of socket walls, significant swelling, or acute infection at the extraction site were excluded. Also, participants with a history of radiotherapy, chemotherapy, immunosuppressive therapy, systemic corticosteroid use, or any anticoagulant therapy within the past 30 days, regular use of NSAIDs, uncontrolled diabetes, presence of malignant tumors, pregnant or lactating women, or those planning pregnancy during the study period were excluded.

### 2.3. Study Groups

The participants were divided into two groups using consecutive allocation with 10 sockets per group:

Group 1 (PRGF group): Sockets were filled with PRGF clot and then covered with PRGF membrane.

Group 2 (SH Group): Sockets were left to heal spontaneously.

### 2.4. Sample Size Calculation

Based on the previous study by Elayah et al. 2023 [32] with measurements of the primary outcome variable (Socket depth) at Baseline (34.9 ± 3.5) and Post-op follow-up of 3 months (29.4 ± 4.3) and considering a power of 80%, attrition of 20%, an effect size of 1.402, alpha set at 0.05 with an allocation ratio of 1, the required sample size of 10 sockets per group was calculated a priori based on the difference between two independent means.

### 2.5. Outcome Measures

Change in the alveolar ridge dimensions was the primary outcome of the study, which was assessed using CBCT. The evaluated parameters consisted of the buccal wall height, measured at the distobuccal, mid-buccal and mesiobuccal points. Alongside, the palatal wall height was assessed at the distopalatal, mid-palatal and mesiopalatal points. The alveolar ridge width was measured at the distal, mid-buccal and the mesial aspects of the socket. The secondary outcomes included the assessment of the CBCT-derived radiographic bone density to check the quality of the bone formation in the healing socket, and Early Healing Index, Postoperative pain (VAS), Soft tissue healing score, and histomorphometric analysis.

### 2.6. Preparation of Plasma Rich in Growth Factors (PRGF)

Blood was collected in 9 mL vacutainers containing 3.8% sodium citrate (BTI-Endoret^®^ System V Centrifuge, Biotechnology Institute, Vitoria, Spain) and then the tubes were centrifuged at 580 g for 8 min using an Endoret System centrifuge (Endoret^®^ System IV, Biotechnology Institute BTI S.L. Miñano, Álava, Spain) [33]. The plasma obtained was then separated into two layers.

F1: Platelet-poor Plasma.

F2: The 2 mL of the plasma column just above the buffy coat.

F2 layer was used to obtain the clot, as it was the fraction richest in platelets, by activating it using PRGF activator (10% calcium chloride). F1 layer was similarly activated to obtain the fibrin membrane [34].

### 2.7. Surgical Procedure

**Figure 1:**
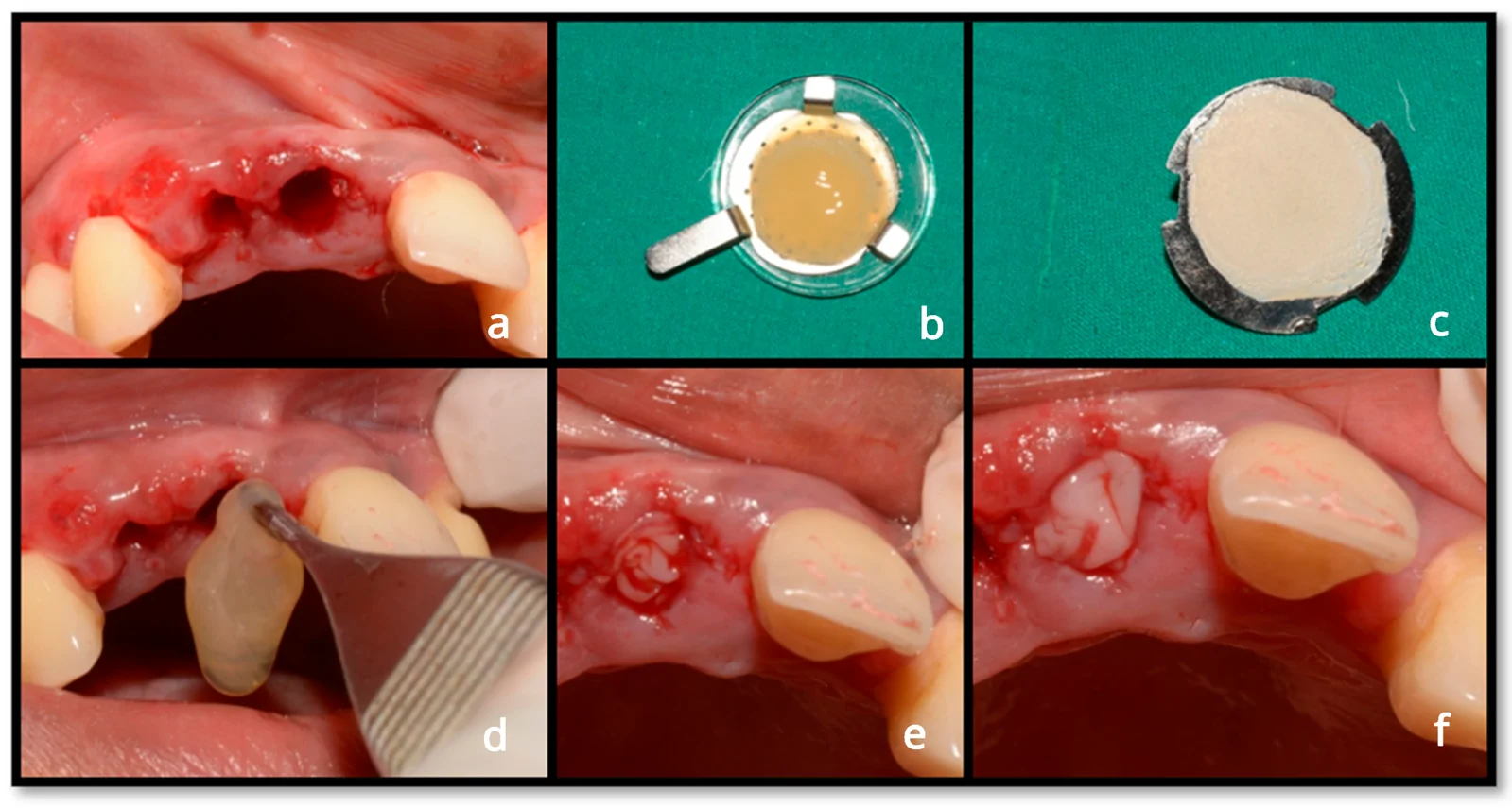
(**a**) Extraction sockets, (**b**) PRGF clot, (**c**) PRGF membrane, (**d**,**e**) Socket packed with PRGF clot, (**f**) Membrane placed.

All the procedures were performed under sterile conditions. 2% lignocaine with 1:100,000 epinephrine was administered to the patients to obtain adequate anesthesia. Atraumatic extraction was carried out in both groups using periotomes and elevators to reduce trauma to the surrounding alveolar bone, buccal cortical plate, and soft tissues. After tooth removal, the socket was debrided meticulously to eliminate granulation tissue and debris. In the PRGF group, after tooth extraction, PRGF clot was packed in the socket, and then the socket was covered with a PRGF membrane (Figure 1). In the SH group, the extraction socket was left to heal without the placement of any biomaterial. A cross-mattress suture was placed using 4-0 non-resorbable silk across the socket to stabilize the clot and facilitate healing. Sutures were removed after 7 days.

### 2.8. Postoperative Care and Follow-Up

A combination of amoxicillin and clavulanic acid (625 mg) and diclofenac (50 mg) with paracetamol (325 mg), administered twice daily for a duration of three days, was prescribed to all the participants. The patients were instructed to maintain a soft diet and were told to avoid chewing on the operated site during the immediate postoperative period. The follow-up was scheduled on the 1st, 3rd, and 7th postoperative day for the assessment of early wound healing, pain, and soft tissue healing indices. Further evaluations were done at 1 month and 3 months postoperatively.

### 2.9. Clinical and Radiographic Assessment

Immediately after tooth extraction, the baseline measurements of the socket were recorded to obtain reference values for further comparison. An impression was made using irreversible hydrocolloid impression material and poured in dental stone to obtain a diagnostic cast. The same process was repeated at 3 months after extraction to obtain a post-healing cast. The dental casts were then scanned using a laboratory scanner (Medit T-Series^®^ Lab Scanner, Medit Corp., Seoul, Republic of Korea). Linear measurements of the mesiodistal and the buccolingual dimensions of the sockets were assessed in Exocad software (Version 3.3 Chemnitz, Darmstadt, Germany) using the STL files. Radiographic dimensional changes of the extraction sockets from baseline to 3 months were assessed using implant planning software (NNT^®^ Viewer, Version 16.7, NewTom^®^, Verona, Italy) using the DICOM datasets obtained from CBCT scans (NewTomGiANO HR^®^, Verona, Italy). The STL and the DICOM files were superimposed to ensure correct image registration. All participants were radiographically evaluated using the same CBCT unit, and a standardized acquisition protocol was followed. The scans were acquired with a voxel size of 0.2 mm, Field of View (FOV) of 80 mm × 50 mm, with a tube voltage of 90 kVp and a tube current of 0.6 mA.

**Figure 2:**
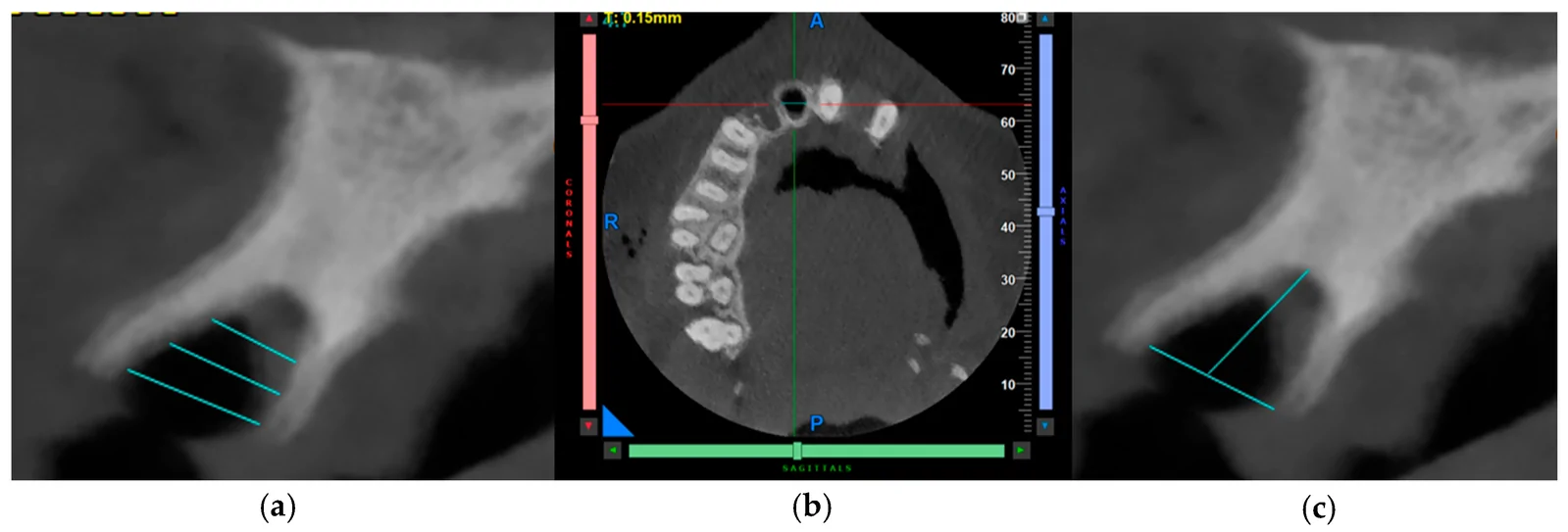
(**a**) Measurements taken of the empty socket using CBCT at the crest, 1 mm from the crest, and 3 mm from the crest; (**b**) mesiodistal dimension; (**c**) socket depth.

**Figure 3:**
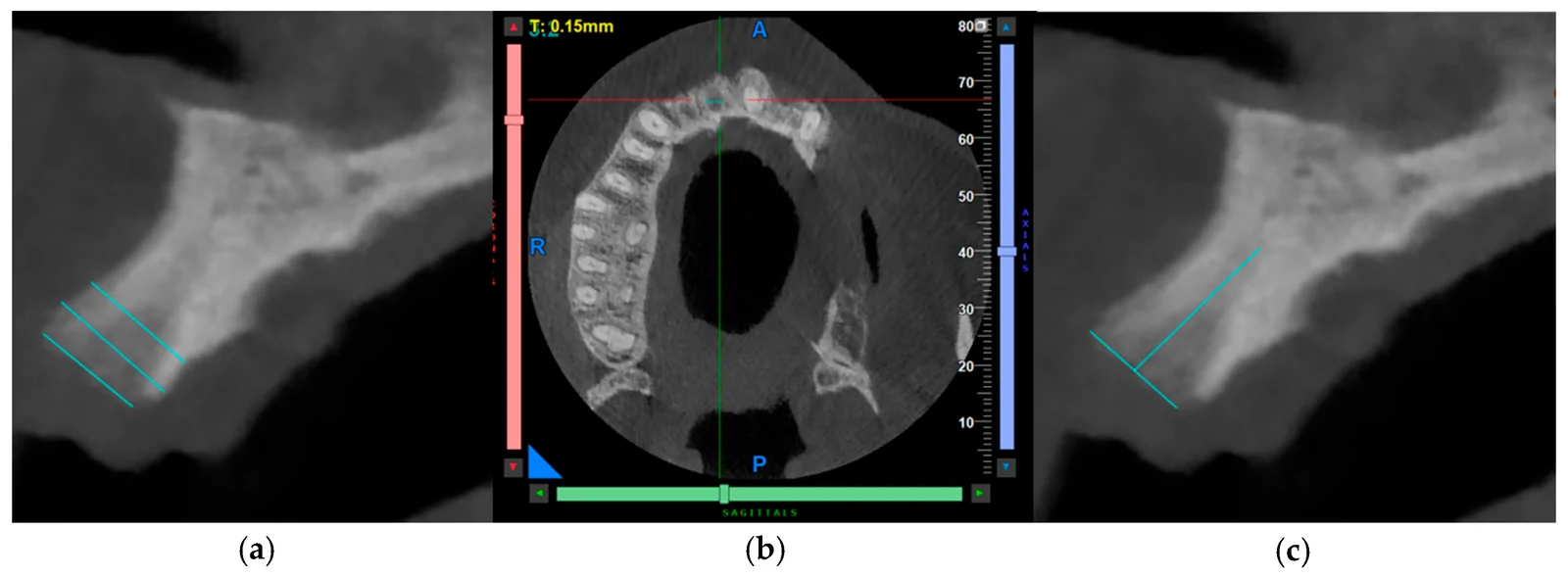
(**a**) Measurements taken of the healed socket using CBCT at the crest, 1 mm from the crest, and 3 mm from the crest; (**b**) mesiodistal dimension; (**c**) socket depth.

Horizontal ridge width was measured at the crestal level and at 1 mm and 3 mm apical to the crest. Socket depth and ridge dimensions were assessed at baseline and at 3 months post-extraction. All measurements were performed under standardized image orientation atthe mid-buccal and mid-palatal aspects by a calibrated examiner to minimize measurement variability. Baseline measurements of the socket were recorded immediately following tooth extraction to establish reference values for further comparison. Socket depth, mesiodistal width, and Buccopalatal width were measured, followed by an impression of the maxillary arch. A diagnostic cast was made and then scanned using a digital scanner to generate a 3D model for further assessment (Figure 2 and Figure 3). A CBCT scan was performed immediately after extraction to evaluate the empty socket to measure the socket dimensions as well as the radiographic bone density. The radiographic bone density was assessed by evaluating the Gray values obtained from the CBCT images. Each socket image was divided into a standardized grid and nine equally distributed points. These values from the nine points were recorded, and the average represented the CBCT-derived radiographic bone density of the socket. CBCT and histo-morphometric assessments were performed by examiners who were aware of the treatment allocation. Blinded outcome assessment was not implemented.

Change values with respect to ridge width and socket depth were calculated for each socket as the 3-month measurement minus the corresponding baseline measurement. The mean and standard deviation of change were calculated from these individual socket-level change values and not from group-level mean values.

### 2.10. Histo-Morphometric Evaluation

After 3 months, a bone core biopsy was obtained from the healed socket using a trephine bur and a stent at the CEJ level of the adjacent teeth, during the initial osteotomy in participants who agreed to implant placement. The biopsy sample was fixed in 10% neutral buffered formalin, processed, and embedded in paraffin. Serial sections of approximately 4–5 µm thickness were prepared using a rotary microtome and stained with hematoxylin and eosin for microscopic assessment.

### 2.11. Statistical Analysis

Statistical analyses were performed using IBM SPSS Statistics for Windows, version 26.0 (IBM Corp., Armonk, NY, USA). The normality of continuous variables was assessed using the Shapiro-Wilk test and inspection of distributional plots. Baseline and change-score comparisons between the groups were performed using the independent-samples *t*-test. Three-month continuous outcomes were compared using analysis of covariance, taking the corresponding baseline measurement as the covariate and treatment group as the fixed factor. Homogeneity of regression slopes was assessed by testing the interaction between treatment group and baseline value. Between-group comparisons of ordinal outcomes at individual time points were performed using the Mann-Whitney U test. Changes over time within each group were assessed using the Friedman test, followed by post-hoc Wilcoxon signed-rank tests with Bonferroni adjustment. A two-sided *p*-value <0.05 was considered statistically significant, with adjusted *p*-values used for multiple comparisons.

## 3. Results

A total of 20 sockets were included in this comparative clinical study, with 10 sockets in the PRGF group and 10 sockets in the SH group. All the participants completed the study protocol and the follow-up period. There was uneventful healing in all the participants, and no postoperative complications were observed.

### 3.1. Baseline Demographic and Clinical Characteristics

The extraction site characteristics and baseline demographics are shown in Table 1. The mean age of the participants in the PRGF group was 44.50 ± 11.92 years and 41.30 ± 12.33 years in the SH group (*p* = 0.562). There was a similar sex distribution between the groups, with 11 females and 9 males. The extracted tooth types were also similar between the groups, with central incisors, lateral incisors and canines being represented in both groups. The SH group had a mean buccal wall thickness of 0.69 ± 0.33 mm and the PRGF group had 0.89 ± 0.14 mm (*p* = 0.094). Overall, both groups were similar at baseline with respect to demographic and extraction characteristics.

### 3.2. Comparison of Ridge Dimensions, Horizontal Ridge Width, Socket Depth, and CBCT Derived Radiographic Bone Density

Table 2 presents the comparison of ridge dimensions, horizontal ridge width, socket depth, and bone density between the SH and PRGF group at baseline and 3 months. Baseline values were similar between the groups for all parameters except bone density, which was significantly higher in the PRGF group (*p* < 0.001). Both groups showed reductions in ridge dimensions and socket depth after 3 months. However, the PRGF group showed significantly better preservation of horizontal ridge width (*p* < 0.001), along with significantly better preservation of socket depth (*p* < 0.001). PRGF group showed a significant increase in bone density when compared with the SH group (*p* < 0.001). Changes in the buccolingual or mesiodistal dimensions were not significant between the groups. Greater preservation of the horizontal ridge width (*p* < 0.001), socket depth (*p* < 0.001), and bone density (*p* < 0.001) in the PRGF group was observed after adjusting for baseline values, while no significant difference was found for buccolingual or mesiodistal dimensions.

### 3.3. Comparison of Soft Tissue Healing and Postoperative Pain

The comparison of early wound healing, postoperative pain, and soft tissue healing between the SH and PRGF groups is shown in Table 3. There was no significant difference in the Early Healing Index between the groups on Day 1 (*p* = 1.000). However, the PRGF group presented significantly better early healing on Day 3 and Day 7 compared with the SH group (*p* = 0.006).

There were significantly lower postoperative pain scores in the PRGF group at all postoperative time points when compared with the SH group (*p* < 0.001).

There was significantly superior soft tissue healing in the PRGF group throughout the follow-up period. At 1 week, the median soft tissue healing score was significantly better in the PRGF group when compared to the SH group (*p* = 0.006). Comparable findings were seen at 1 month and 3 months, signifying enhanced soft tissue healing in the PRGF group.

The post-hoc analysis was carried out for soft tissue healing and postoperative pain scores at different time points and presented as Supplementary Tables S1 and S2.

### 3.4. HistomorphometricAssessment

Quantitative histomorphometricdata were available for a subset of participantswho agreed toimplant placement and are presented in Table 4. There were no significant intergroup differences in bone biopsy length, new bone formation, or connective/fibrovascular tissue.

## 4. Discussion

The resorption of the alveolar ridge after tooth extraction remains a major clinical challenge, especially in the anterior maxillary region, where both functional and aesthetic demands are high [3,35]. This occurrence has been seen as substantial dimensional changes that follow tooth extraction [36]. Participants were enrolled consecutively according to eligibility criteria and allocated to the PRGF and SH groups using consecutive allocation. Formal randomization and allocation concealment were not implemented. The study was therefore classified as a prospective, non-randomized comparative clinical study. This study was designed to check the clinical effectiveness of PRGF as a biologically driven option for alveolar ridge preservation in comparison to SH. The results of this study show that PRGF has an advantageous influence on post-extraction healing, mainly in terms of horizontal ridge preservation, bone density, soft tissue healing, and postoperative comfort.

Baseline characteristics between the PRGF group and the SH group were similar, except for CBCT-derived radiographic bone density assessed post-extraction, granting a valid intergroup comparison. This is harmonious with the methodology recommended by Araújo and Lindhe, who highlighted the importance of baseline standardization in studies checking the alveolar bone dynamics [37]. Both groups showed dimensional changes in the alveolar ridge after extraction, which matches the biological principles mentioned by Araújo and Lindhe, who showed that post-extraction bone remodeling is essentially due to the resorption of the bundle bone. Both buccolingual and mesiodistal dimensions showed a reduction in this present study, but there was no statistically significant difference between PRGF and SH [38]. Tan et al., in their systematic review, have reported similar observations that conclude that the dimensional changes are inevitable [39]. CBCT measurements demonstrated a statistically significant preservation of the horizontal ridge width in the PRGF group at all the assessed levels. Avila-Ortiz et al. highlighted the importance of the 3D radiographic assessment in precisely detecting post-extraction dimensional changes [35]. Similar results have been demonstrated in existing literature evaluating platelet concentrates for alveolar ridge preservation, where improved dimensional stability and reduced post-extraction collapse were seen [40].

The benefits of PRGF lie in its ability to increase the natural healing cascade and provides a fibrin scaffold enhanced with growth factors like PDGF, TGF-β and VEGF, which promote angiogenesis and osteogenesis [41,42,43]. This mechanism has been shown by Anitua et al., who showed the regenerative potential of PRGF in bone healing. PRGF facilitates intrinsic tissue regeneration, unlike graft materials that mainly act through osteoconduction [44].

This study also reported a significant increase in CBCT-derived radiographic bone density in the PRGF group. These findings match the reports published by Del Fabbro et al., who concluded that the autologous platelet concentrates may increase the bone maturation and mineralization [45]. Moreover, the absence of any residual graft material in PRGF-treated sites supports the concept of biological regeneration. Socket depth preservation was significantly greater in the PRGF group, backing up the beneficial effect of PRGF on maintaining the post-extraction socket structure during healing [46]. Histomorphometric analysis supported these findings by showing no significant difference with respect to the percentage of new bone formation and connective tissue/fibrovascular compared to the SH group, and indicated active bone regeneration and favorable tissue maturation. These findings are similar to the observations of previous studies, which suggested the regenerative potential of autologous platelet concentrates [47] in increasing tissue healing and bone formation [22,27,28,29,40,42].

The PRGF group showed statistically significant soft tissue healing at all time intervals in comparison to the SH group. This can be credited to the sustained release of growth factors along with the formation of a stable fibrin matrix [48,49]. Better soft tissue healing is essential in the anterior maxilla due to its aesthetic significance. In the PRGF group, the postoperative pain was significantly lower, which can be explained by its anti-inflammatory properties. Anitua et al. concluded that PRGF can modulate inflammation and reduce postoperative discomfort, hence improving the patient experience. Regardless of these advantages, PRGF did not completely prevent alveolar ridge resorption. This is consistent with Tan et al. and Vignoletti et al., who suggested that no currently available technique can eliminate post-extraction bone loss but can only decrease the extent [39,50].

There are some limitations of this study, such as a relatively small sample size and a short follow-up period of 3 months. Given the non-randomized design, selection bias cannot be excluded. Although baseline-adjusted analyses were performed for continuous outcomes, residual confounding may remain, particularly for bone-density outcomes because baseline bone density differed between groups. The significant baseline difference in CBCT derived radiographic bone density between the groups represents a potential confounding factor and may have influenced the observed changes over time; therefore, the bone density findings should be interpreted cautiously. Additionally, blinded outcome assessment was not performed. This may have introduced detection or observer bias, particularly for radiographic and histo-morphometric measurements. This limitation should also be considered when interpreting the findings. Histo-morphometricsamples were obtained from a limited number of participants due to the clinical constraints of patients opting for fixed and partial prostheses, thereby limiting biopsy specimens.Therefore, the histo-morphometric findings should be interpreted as exploratory. Although the socket-level audit reproduced the reported values, the small sample size and low dispersion of ridge-width change values limit generalisability. These findings should therefore be considered preliminary and require confirmation in larger randomized studies with independent measurement validation. Future research with a larger sample size and longer follow-up duration is needed to validate these findings. Even with these limitations, this study suggests that PRGF is a favorable biomaterial for alveolar ridge preservation. It provides advantages in terms of improved bone quality, reduced resorption, enhanced soft tissue healing, and reduced postoperative discomfort without the requirement of a particulate graft material.

## 5. Conclusions

PRGF showed a promising short-term effect on post-extraction healing in the anterior maxilla when compared to spontaneous healing. PRGF demonstrated significantly improved preservation of horizontal ridge dimensions, increased CBCT-derived radiographic bone density, improved soft tissue healing, and reduced postoperative pain. The use of PRGF efficiently reduced the extent of alveolar bone resorption and showed similar histo-morphometric findings to those of SH, even though the dimensional changes of the alveolar ridge were not completely prevented. Within the limitations of this study, PRGF may be considered a favorable approach for alveolar ridge preservation, especially in the anterior maxilla, where both hard and soft tissue outcomes are important.

## Figures and Tables

**Table 1:** Baseline Demographic and Clinical Characteristics of Study Participants.

Characteristic	SH (*n* = 10)	PRGF (*n* = 10)	Total (*n* = 20)
Age (years)	41.30 ± 12.33	44.50 ± 11.92	42.90 ± 11.92
Sex, (*n* %)			
Female	5 (50.0)	6 (60.0)	11 (55.0)
Male	5 (50.0)	4 (40.0)	9 (45.0)
Tooth type, (*n* %)			
Central incisor	3 (30.0)	4 (40.0)	7 (35.0)
Lateral incisor	3 (30.0)	3 (30.0)	6 (30.0)
Canine	4 (40.0)	3 (30.0)	7 (35.0)
Buccal wall thickness (mm)	0.69 ± 0.33	0.89 ± 0.14	0.79 ± 0.27

SH, Spontaneous Healing; PRGF, Plasma Rich in Growth Factors.

**Table 2:** Comparison of Ridge Dimensions, Horizontal Ridge Width, Socket Depth, and Bone Density between SH and PRGF groups.

Outcome	SH Baseline	SH 3 Months	SH Change	PRGF Baseline	PRGF 3 Months	PRGF Change	*p* Value * Comparison of Change	Adjusted Mean Difference (PRGF—SH), 95% CI	*p*-Value † (ANCOVA)
Model buccolingual dimension (mm)	6.80 ± 1.48	4.81 ± 0.84	−1.99 ± 0.97	6.47 ± 1.93	5.26 ± 1.05	−1.21 ± 1.46	0.175	0.58 (−0.07 to 1.23)	0.076
Model mesiodistal dimension (mm)	5.94 ± 2.11	4.61 ± 1.35	−1.33 ± 0.92	5.90 ± 1.94	4.96 ± 1.15	−0.94 ± 1.13	0.415	0.37 (−0.15 to 0.88)	0.151
Horizontal ridge width at crest (mm)	5.98 ± 0.26	4.13 ± 0.21	−1.85 ± 0.08	5.99 ± 0.26	4.89 ± 0.24	−1.10 ± 0.04	<0.001	0.76 (0.71 to 0.80)	<0.001
Horizontal ridge width at 1 mm (mm)	6.58 ± 0.28	4.93 ± 0.22	−1.64 ± 0.08	6.61 ± 0.27	5.69 ± 0.26	−0.92 ± 0.04	<0.001	-	-
Horizontal ridge width at 3 mm (mm)	7.47 ± 0.28	5.86 ± 0.25	−1.61 ± 0.08	7.50 ± 0.27	6.61 ± 0.27	−0.90 ± 0.04	<0.001	0.72 (0.66 to 0.77)	<0.001
Socket depth (mm)	10.07 ± 0.25	9.20 ± 0.23	−0.87 ± 0.07	10.09 ± 0.23	9.47 ± 0.21	−0.62 ± 0.03	<0.001	0.25 (0.20 to 0.30)	<0.001
CBCT derived Radiographic Bone density	431.2 ± 17.1	511.8 ± 16.9	80.6 ± 5.1	532.7 ± 16.6	661.2 ± 17.1	128.5 ± 3.4	<0.001 †	50.44 (36.51 to 64.38)	<0.001

Values are presented as mean ± standard deviation. Change was calculated as the 3-month value minus the baseline value. * Unpaired *t*-test; † ANCOVA; *p* < 0.05. Standard ANCOVA was not applied to horizontal ridge width at 1 mm because the group × baseline interaction was significant (*p* = 0.020).Adjusted mean differences are reported as PRGF minus SH.

**Table 3:** Comparison of Soft Tissue Healing Scores and Postoperative Pain Scores Between SH and PRGF Groups.

Outcome	Time Point	SH Median (IQR)	PRGF Median (IQR)	Mann-Whitney U	*p*-Value *
Early Healing Index	Day 1	3 (3–3)	3 (3–3)	50	1.000
	Day 3	2.5 (2–3)	1.5 (1–2)	12.5	0.006
	Day 7	2 (2–2.25)	1 (1–1.25)	13	0.006
Postoperative pain (VAS Score)	Day 1	4 (4–5)	2.5 (2–3)	0	<0.001
	Day 3	2.5 (2–3)	1 (1–1.25)	5	<0.001
	Day 7	1 (0–1)	0 (0–0)	15	0.001
Soft tissue healing score	1 week	3 (2–3)	3 (3–4)	18	0.006
	1 month	3 (3–4)	4 (4–5)	12	0.003
	3 months	4 (4–4)	5 (5–5)	5	<0.001

Values are presented as median (25th–75th percentile). SH, spontaneous healing; PRGF, plasma rich in growth factors; IQR, interquartile range; VAS, Visual Analog Scale. * Mann-Whitney U test (adjusted within each outcome across its three time points using the Holm method).

**Table 4:** Quantitative histomorphometriccomparison between the SH (*n* = 6) and PRGF (*n* = 5) groups.

Parameter	SH Mean ± SD	SH Median (IQR)	PRGF Mean ± SD	PRGF Median (IQR)	95% CI of the Difference	Mann-Whitney U	*p*-Value
Bone biopsy length (mm)	2.67 ± 0.52	3.00 (2.25–3.00)	3.00 ± 0.71	3.00 (3.00–3.00)	0.33 (−0.56 to 1.23)	19.00	0.454
New bone formation (%)	26.50 ± 2.88	26.50 (24.50–27.75)	26.00 ± 3.39	27.00 (23.00–28.00)	−0.50 (−4.93 to 3.93)	13.5	0.854
Connective/fibrovascular tissue (%)	56.17 ± 3.82	56.50 (54.50–59.25)	53.80 ± 6.46	55.00 (54.00–57.00)	−2.37 (−10.32 to 5.59)	12	0.644

SH, spontaneous healing; PRGF, plasma rich in growth factors; CI, confidence interval; IQR, interquartile range; Mann-Whitney U test, *p* ≤ 0.05.

## Data Availability

The analyzed datasets are available from the corresponding author on request.

## Supplementary Materials

The following supporting information can be downloaded at: https://www.mdpi.com/article/10.3390/jfb17090452/s1, Table S1: Within-group changes over time, Table S2: Post-hoc pair-wise comparisons.

## Abbreviations

The following abbreviations are used in this manuscript:

APCs	Autologous Platelet Concentrates
CBCT	Cone Beam Computed Tomography
CI	Confidence Interval
DICOM	Digital Imaging and Communications in Medicine
FOV	Field of View
IBM	International Business Machines
IQR	Interquartile Range
NSAIDs	Nonsteroidal Anti-Inflammatory Drugs
PDGF	Platelet-Derived Growth Factor
PRGF	Plasma Rich in Growth Factors
SH	Spontaneous Healing
SPSS	Statistical Package for the Social Sciences
STL	Standard Tessellation Language
TGF-β	Transforming Growth Factor Beta
VEGF	Vascular Endothelial Growth Factor
VAS	Visual Analog Scale
