# Supplementary material for: Clinical and Radiographic Evaluation of Plasma Rich in Growth Factors (PRGF) for Alveolar Ridge Preservation in the Anterior Maxilla
Source: J Funct Biomater. 2026 Sep 7;17(9):452. doi: 10.3390/jfb17090452 (PMC13608091; doi:10.3390/jfb17090452)
Supplement: Supplementary file 1 [file jfb-17-00452-s001.zip › jfb-4491334-supplementary.pdf]

Supplementary Tables

Supplementary Table S1. Within-group changes over time

Outcome	Group	Time point 1	Time point 2	Time point 3	Friedman χ^2	df	*P value
**Early Healing Index**	SH	3 (3-3)	2.5 (2-3)	2 (2-2.25)	12.56	2	0.002
**Early Healing Index**	PRGF	3 (3-3)	1.5 (1-2)	1 (1-1.25)	18.727	2	<0.001
**Postoperative pain**	SH	4 (4-5)	2.5 (2-3)	1 (0-1)	20	2	<0.001
**Postoperative pain**	PRGF	2.5 (2-3)	1 (1-1.25)	0 (0-0)	20	2	<0.001
**Soft-tissue healing score**	SH	3 (2-3)	3 (3-4)	4 (4-4)	16.545	2	<0.001
**Soft-tissue healing score**	PRGF	3 (3-4)	4 (4-5)	5 (5-5)	17.176	2	<0.001

Values at each time point are presented as median (25th-75th percentile).
Time points: Early Healing Index and postoperative pain: Day 1, Day 3, and Day 7; Soft-tissue healing score:1 week, 1 month, and 3 months.
SH, spontaneous healing; PRGF, plasma rich in growth factors; df, degrees of freedom.
** Friedman test.* P≤0.05

Supplementary Table S2. Post-hoc pairwise comparisons

Outcome	Group	Pairwise comparison	Z	*P value (Bonferroni-adjusted)	Interpretation
**EHI**	SH	Day 1 vs Day 3	-2.24	0.076	Not significant
**EHI**	SH	Day 1 vs Day 7	-2.71	0.02	Significant
**EHI**	SH	Day 3 vs Day 7	-2.00	0.137	Not significant
**EHI**	PRGF	Day 1 vs Day 3	-2.88	0.012	Significant
**EHI**	PRGF	Day 1 vs Day 7	-2.97	0.009	Significant
**EHI**	PRGF	Day 3 vs Day 7	-1.73	0.25	Not significant
**PP**	SH	Day 1 vs Day 3	-2.91	0.011	Significant
**PP**	SH	Day 1 vs Day 7	-2.92	0.011	Significant
**PP**	SH	Day 3 vs Day 7	-2.97	0.009	Significant
**PP**	PRGF	Day 1 vs Day 3	-2.92	0.011	Significant
**PP**	PRGF	Day 1 vs Day 7	-2.88	0.012	Significant
**PP**	PRGF	Day 3 vs Day 7	-2.97	0.009	Significant
**STHS**	SH	1 week vs 1 month	-2.27	0.069	Not significant
**STHS**	SH	1 week vs 3 months	-2.88	0.012	Significant
**STHS**	SH	1 month vs 3 months	-2.65	0.024	Significant
**STHS**	PRGF	1 week vs 1 month	-2.64	0.025	Significant
**STHS**	PRGF	1 week vs 3 months	-2.89	0.012	Significant
**STHS**	PRGF	1 month vs 3 months	-2.45	0.043	Significant

EHI, Early Healing Index; STHS, PP- Postoperative pain; Soft-tissue healing score.
*Wilcoxon Signed Ranks Test. $P \leq 0.05$
